# King Edward's Hospital Fund for London

**Published:** 1904-04-02

**Authors:** 


### Editor's Letter-Box.

[Our correspondents are reminded that prolixity is a great bar to publication, and that brevity of style and conciseness of statement greatly facilitate early insertion.]

### KING EDWARD'S HOSPITAL FUND FOR LONDON.

Mr. Hugh C. Smith, Chairman of the Executive Committee, writes :—The President, his Royal Highness the Prince of Wales, in his speech at Marlborough House on March 8th last, announced that a large sum, estimated to produce £4,600 a year, had been promised by a friend if a further sum of double that amount were obtained by the end of this year, in order to raise the annual income of this fund, from investments and other permanent sources, to £50,000 a year.

The object of the anonymous donor is to enable the Council to make their distributions to the hospitals without again drawing upon reserves as last year, when the sum of £18,751 9s. was thus reluctantly appropriated to complete the grants.

We have great pleasure in stating that we have already received from "G. W. C." a donation of £1,000 towards this object, besides a legacy of £10,000 from Mr. F. S. Massy Dawson, the executor of the late Mr. Richard Hawkins Beauchamp, which we propose devoting to the same purpose.

We therefore venture to make this appeal in the hope that the requisite sum will be provided during the present yeart to enable this munificent offer to be secured.

We shall be glad to give further information to any who desire it, and ask that all communications may be addressed either to myself or to the Hon. Secretaries of King Edward's Hospital Fund for London, 81 Cheapside, E.C.

